# The Effects of Stress on Tourniquet Application and CPR Performance in Layperson and Professional Civilian Populations

**DOI:** 10.1177/00187208211021255

**Published:** 2021-05-26

**Authors:** Marc Friberg, Carl-Oscar Jonson, Victor Jaeger, Erik Prytz

**Affiliations:** 1 4566 Linköping University, Sweden

**Keywords:** tourniquet, stress, first aid, laypeople, first responders

## Abstract

**Objective:**

The purpose of this study was to compare laypeople’s and professional first responders’ ability to perform tourniquet application and cardiopulmonary resuscitation (CPR) during calm and stressful circumstances.

**Background:**

Life-threatening bleeding is a major cause of death that could be prevented by fast and appropriate first aid interventions. Therefore, laypeople are now being trained in bleeding control skills, transforming them from bystanders to immediate responders. However, critics have questioned whether laypeople are able to perform during more stressful conditions.

**Method:**

Twenty-four laypersons and 31 professional first responders were tested in two conditions: a calm classroom scenario and a stressful scenario consisting of paintball fire and physical exertion. Stress and workload were assessed along with task performance.

**Results:**

The experimental manipulation was successful in terms of eliciting stress reactions. Tourniquet application performance did not decline in the stressful condition, but some aspects of CPR performance did for both groups. First responders experienced higher task engagement and lower distress, worry and workload than the laypeople in both the calm and stressful conditions.

**Conclusion:**

Stress did not affect first responders and laypeople differently in terms of performance effects. Stress should therefore not be considered a major obstacle for teaching bleeding control skills to laypeople.

**Application:**

Tourniquet application can be taught to laypeople in a short amount of time, and they can perform this skill during stress in controlled settings. Concerns about laypeople’s ability to perform under stress should not exclude bleeding control skills from first aid courses for civilian laypeople.

## Introduction

Trauma is the third leading cause of death in the United States (Rhee et al., 2014). Among these, uncontrolled bleeding is a major cause of death that could have been prevented with appropriate first aid interventions (Kleber et al., 2013; Pelinka et al., 2004). A model by Tjardes and Luecking (2018) suggests that the window of opportunity to save an individual with massive extremity bleeding could be as little as 3 min. Therefore, the people on the scene of the trauma incident are a crucial first link in the trauma chain of survival (Bakke et al., 2015). The U.S. Hartford Consensus recognized the potential in training, empowering, and equipping bystanders and turning them into immediate responders to save lives by applying hemorrhage control devices to traumatic bleedings (Jacobs et al., 2013).

In civilian prehospital settings, hemorrhage control can be achieved by applying direct pressure to the wound or by applying a tourniquet to a wounded extremity. Much of the current knowledge on hemorrhage control originates from the military domain (Kauvar et al., 2018). Therefore, there has been a debate over whether it is possible to transfer this knowledge and experiences to the civilian domain. Specifically, there has been a point of contention whether civilian laypeople are capable of performing hemorrhage control techniques, most commonly tourniquet application, outside educational settings. This is reflected in a recent consensus publication outlining a prioritized research agenda for prehospital hemorrhage control by laypeople, where one of the research questions is “Can/should bleeding control be added to existing first-aid training?” (Goralnick et al., 2020).

There is evidence to show that it is possible to teach laypeople tourniquet usage with short interventions (e.g., Baruch et al., 2017; Hegvik et al., 2017; Jacobs & Burns, 2015; Sidwell et al., 2018). Goolsby, Strauss-Riggs, et al. (2018), for instance, showed that a web-based education together with just-in-time instructions is sufficient for 75% of the participants to correctly apply a tourniquet. However, other studies show very low success rates for trained laypeople, for example, 25% in Baruch et al. (2017).

One potential reason for the low performance of laypeople that has been mentioned is stress (Goolsby, Strauss-Riggs, et al., 2018). There are several sources of stress in a situation that features a life-threatening bleeding from a trauma. For instance, there could be threats to the person’s own physical safety in situations where the trauma was caused by a yet ongoing danger as well as other adverse environmental conditions such as noise, heat, or cold. There are also less tangible sources of stress, such as perceived lack of control, high cognitive workload, and frustration with not being able to help more (Bohström et al., 2017; Kindness et al., 2014). Fear of losing the patient, or even a friend or colleague, is another relevant stressor, as are feelings of loneliness and isolation for immediate responders (Kindness et al., 2014). For professional first responders, stressors such as having insufficient information about the situation, feelings of personal shortcomings and inadequacy, and worry over lack of resources can also be present (Bohström et al., 2017).

A hypothesis that has been suggested is therefore that laypeople, when tested in more realistic conditions, would perform worse than in a calm classroom environment due to increased stress. This hypothesis also implies that civilian layperson immediate responders would underperform in real life situations, which by their nature should be more stressful than educational settings. However, few studies have examined this explicitly and none in a civilian laypeople context.

Sanak et al. (2018) and Schreckengaust et al. (2014) both studied performance of bleeding control interventions in stressful environments, but for trained military populations rather than civilian populations. Sanak et al. (2018) only notes that trained military personnel had more difficulties in applying tourniquets during simulated close combat situations without further elaboration. Schreckengaust et al. (2014), however, compared the performance in a classroom environment and in a stressful, simulated combat scenario. They showed that the time to apply the tourniquet increased in the stressful scenario that featured an outdoors obstacle course and paintball fire, and they noted a decrease in application quality although this was not significant. A limitation, however, is that they did not include any measure of experienced stress in the experimental design. It is therefore unclear if the performance differences come from a stress reaction or from differences in the physical setting.

There is a consensus in current human factors literature that stress cannot be inferred only based on the presence of stressors in the environment (e.g., Hancock & Warm, 1989; Hockey, 1997; Matthews, 2001). Lazarus and Folkman (1984) describe stress as a subjective state experienced when a person appraises that the demands exceed their coping ability. This implies that the same stimuli can be appraised differently by different people. Matthews (2001) has further argued that stress is a multidimensional construct. Performance decrements may manifest when a person’s physiological or behavioral coping strategies are insufficient (Hancock & Warm, 1989). Thus, despite the efforts of Schreckengaust et al. (2014) and other educators (Goolsby, Strauss-Riggs, et al., 2018; Goolsby, Jacobs, et al., 2018), it remains an open question to which extent stress impacts laypeople’s tourniquet performance.

The aim of the current study was therefore to investigate the effects of stress on tourniquet performance in a civilian laypeople population. The study is a methodological replication of Schreckengaust et al. (2014) with some key differences. This study includes a comparison between laypeople and professional first responders, whereas Schreckengaust and colleagues looked at only one training cohort. Further, this study uses a civilian sample rather a military sample, as in Schreckengaust et al. (2014). The training intervention was reduced from 3 days to a brief 20-min training intervention more in line with the typical educational interventions for civilian populations. Further, the experimental setup included validated measures of stress, both physiological and subjective, as well as performance. Finally, a common first aid intervention currently taught to civilians, namely cardiopulmonary resuscitation (CPR), was included as a comparative task.

## Method

### Participants

Participants were recruited from a university in south central Sweden and from the surrounding municipalities. A total of nine women and 46 men aged between 18 and 63 (*M* = 34.02, SD = 11.26) participated in this study. The study sample consisted of one group of 24 laypersons (six women, 18 men) and one group of 31 professional first responders (six women, 25 men). Of these 31, 20 were rescue service workers and 11 emergency medical service workers. The mean number of years of work experience for professionals in the rescue service was 11.6 years, and 13.3 years for the emergency medical services. None of the laypeople had experience as first responders or other medical training. During the recruitment, information about the purpose of the study and the obstacle course and paintball firing was provided. Participants did not receive any compensation for participation in this study. Speaking Swedish and a minimum age of 18 were set as inclusion criteria. This research complied with the tenets of the Declaration of Helsinki and was approved by the Swedish Ethical Review Authority, approval number 2018/305-31. Informed consent was obtained from each participant.

### Procedure

**Figure 1:**
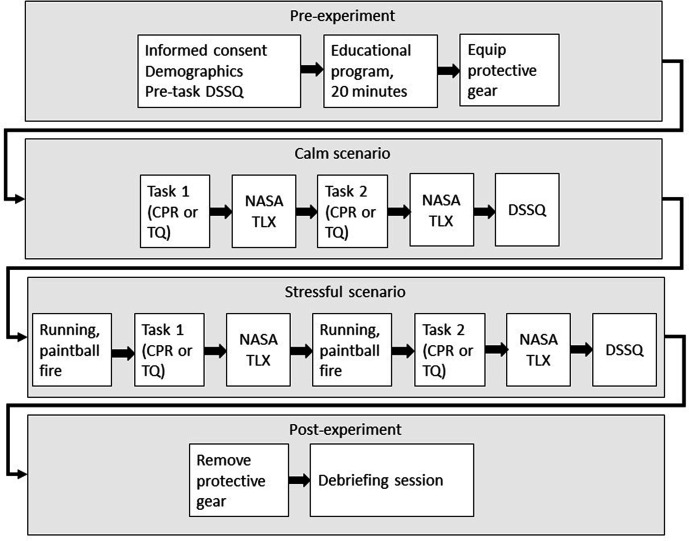
Experimental timeline. DSSQ is the Dundee Stress State Questionnaire, NASA Task Load Index (TLX) is the workload questionnaire, and the abbreviation TQ is used to denote the tourniquet application task.

The experiment was divided into four phases: pre-experiment, calm scenario, stressful scenario, and post-experiment. An overview is shown in Figure 1. In the first, pre-experiment phase, participants were welcomed and filled out a general questionnaire for demographics, a baseline stress questionnaire, and a consent form. They then took part of an educational program on first aid. The educational program consisted of a 7-min video about prehospital hemorrhage control followed by practical tourniquet application exercises and a live demonstration of CPR on a simulation manikin. The practical tourniquet applications were performed three times by the participants: on their own leg, on another participant’s leg, and on their own arm.

Participants were then, one by one in turn, given protective equipment and led into an adjacent classroom for phase two, the calm scenario. The scenario was divided into two stations: CPR and tourniquet application. Each station consisted of two plywood sheets formed into a L-shaped configuration with a manikin placed on the “inside” of the L. Participants were given instructions and directed to complete the tasks. For the CPR task, the participants were asked to perform CPR on the manikin for 90 s. The participants performed compressions-only CPR as they wore a protective facemask. The tourniquet application task was to apply a tourniquet to the leg of a manikin. Task order was counterbalanced across participants. The participants completed a workload questionnaire after each station and an additional stress state questionnaire after having completed both stations.

The third phase, the stressful scenario, was conducted in an adjacent exercise hall. Two stations were placed in a similar configuration as the calm scenario. Additionally, two plywood sheets standing on the long edge were used as covers in front of the stations. Participants were instructed to run between the two covers, which were approximately 10 meters apart, at the command of an instructor. The participants were subject to paintball fire and had to lay down behind the covers. The instructor ran the participants between the two covers eight times and then ordered them to one of the two stations to perform the task. There was no paintball fire during the task. The running procedure was then repeated, until the instructor again ordered the participant to the other station. The participants again completed a workload questionnaire after each station. The participants filled out the final stress state questionnaire after completing the two stations, and the experiment was concluded with a short debriefing session.

### Material

In total, four questionnaires were issued to the participants throughout the experiment: the Dundee Stress State Questionnaire (DSSQ; Matthews, 2016), the raw version of NASA Task Load Index (NASA-TLX; Hart & Staveland, 1988), a demographic pre-experiment questionnaire, and a post-experiment questionnaire. The DSSQ measures stress on three dimensions: task engagement, distress, and worry. Briefly, task engagement reflects the participants focus and engagement with the task at hand, distress reflects negative emotions and perceived loss of control, whereas worry reflects intrusive, unwanted thoughts about one’s self-image. The NASA TLX consists of six subscales: mental demands, temporal demands, physical demands, performance, frustration, and effort. For the current study, the global mean of these subscales was used. The demographic pre-experiment questionnaire consisted of questions regarding age, sex, experience from emergency services, rescue services, military and the police, and previous bleeding control training. The post-experiment questionnaire contained some knowledge assessment items and questions regarding the experimental manipulations. The post-experiment questionnaire is not analyzed further in the current study.

The Combat Application Tourniquet (CAT) was used during the experiment. Tourniquet application performance was measured using a slightly revised assessment checklist as used in previous studies (Lowndes et al., 2017). Each application was given a score between 1 and 8 based on tourniquet placement, strap and windlass tightness, notation of time, and how secure the tourniquet was for transportation without risk of sliding off. Two GoPro cameras were mounted at each task station, recording the task performance from different angles. Two raters, including one experienced Tactical Combat Casualty Care instructor, analyzed the video material for task performance according to the assessment template, as well as recorded time to complete application and time to bleeding control, in seconds. Performance time was recoded from the moment the participant touched the tourniquet and ended, for the total time, when the participant reported “Done” to the instructor. Time to bleeding control was stopped when the participant had secured the windlass in the clip after tightening.

CPR was performed on the Laerdahl Little Anne QCPR simulation manikin, and performance was recorded using the QCPR instructor app (Laerdhal Medical AS, version 3.4.11). Compressions were continuously performed for 90 s, and three measures of performance were recorded: average compression rate, percentage of compressions with sufficient depth, and percentage of compressions with sufficient release.

Heart rate (HR) and heart rate variability (HRV) data were recorded using a commercial Polar H10 chest strap pulse recorder during the calm and stressful scenario. The pulse recorder was placed, by the participant, directly on the skin below the sternum. In order to increase conductivity to the skin, the recorder was damped with a piece of cloth. The data were captured using the Elite HRV app (Perrotta et al., 2017) and analyzed using the Kubios Standard (version 3.1.0.1) software. An autoregressive power spectral density model was used to extract the heart beat-to-beat interval (R-R) data. Artifacts were corrected using a threshold-based artifact correction algorithm. All R-R values were compared with a local average interval, and if the value varied more than a fixed threshold value, predetermined to .45 s, the R-R value was considered an artifact and interpolated using a cubic spline interpolation (Tarvainen et al., 2014). Measures used for this study were average HR and HRV in the low frequency (LF; 0.04–0.15 Hz) and high frequency (HF; 0.15–0.4 Hz) power band. Generally, stress is associated with an increase in LF power and a decrease in HF power (Kim et al., 2018). HF to LF ratio is also sometimes used; however, there is some evidence that LF power may be very low during periods of very high stress which could make ratio comparisons misleading (von Rosenberg et al., 2017). A secondary task in the form of mental arithmetic was also performed concurrently with the tourniquet application and CPR tasks for both conditions. The results of this task are however not analyzed further in the current study.

A compressed air powered Angel A1 Fly paintball marker was used during the stressful scenario. Muzzle velocity was set to circa 90 m/s, and ammunition was of reball type cal. 68. Participants wore an eye-fan face protection mask as well as a suspensor. This equipment was mandatory during both the calm and stressful scenario. Additional protective gear for the knees and elbows was available but not required.

### Analyses

The independent variables were Condition (calm or stressful), Task (CPR or tourniquet), and Group (laypeople or first responders). For the DSSQ, the condition variable also included the pre-experiment baseline. The dependent variables are listed in Table 1.

**Table 1:** Dependent and Independent Variables

Dependent Variables	Independent Variables	Measures
Tourniquet performance	Condition: Calm, Stressful, Group: Laypeople, First Responders	Time to bleeding control
		Total application time
		Checklist score
CPR performance	Condition: Calm, Stressful Group: Laypeople, First Responders	Depth
		Release
		Compression frequency
Stress (subjective)	Condition: Baseline, Calm, Stressful Group: Laypeople, First Responders	Task Engagement
		Distress
		Worry
Stress (physiological)	Condition: Calm, Stressful Group: Laypeople, First Responders	HR
		LF HRV HF HRV
Workload	Condition: Calm, Stressful Group: Laypeople, First Responders Task: CPR, Tourniquet	NASA TLX

*Note*. CPR = cardiopulmonary resuscitation; HR = heart rate; HRV = heart rate variability; NASA TLX = NASA Task Load Index.

The performance measures for tourniquet application and CPR were analyzed using 2 × 2 split-plot ANOVAs with Condition (calm, stressful) as the within-subjects variable and Group (laypeople, first responders) as the between-subjects variable. The physiological response variables were analyzed in the same way. The three DSSQ dimensions were analyzed using 3 × 2 split-plot ANOVAs with Condition (baseline, calm, stress) as the within-subjects variable and Group (laypeople, first responders) as the between-subjects variable. Significant effects of condition were followed up using planned contrasts, by first comparing the baseline condition to the task performance conditions (the calm and stressful conditions combined) and second comparing the calm and stressful conditions. The NASA TLX grand means were analyzed using 2 × 2 x 2 split-plot ANOVAs with Condition (calm, stress) and Task (CPR, tourniquet application) as the within-subjects variables and Group (laypeople, first responders) as the between-subjects variable.

Huynh-Feldt corrections were used to correct for violations of sphericity and the corrected F-strings are reported where applicable. Nonparametric tests were used when Levene’s test of homogeneity of variance was significant. This was typically the case for the task performance measures, where the novice group displayed greater variance than the professional first responder group.

## Results

### Performance Measures

For the tourniquet performance, there was a significant effect of group on the time to bleeding control such that the professional first responders were significantly faster (median = 45.0, SD = 10.47) compared with the laypeople (median = 65.50, SD = 22.71) across conditions see Table 2 for this and other significant statistical tests. There was no effect of condition, *p* = .938, or group by condition interaction, *p* = .516. The same pattern held for the total application time, where the first responders again were faster (median = 57.0, SD = 12.16) compared with the novices (median = 77.0, SD = 25,44). There was also a significant effect of condition on the tourniquet checklist scores, Wilcoxon signed rank W(55) = 289.50, z = 2.043, *p* = .041. The effect was such that the participants overall scored higher in the stressful condition (median = 8.0, SD = .96) than the calm condition (median = 7.0, SD = .93). There was no effect of group, *p* = .126.

**Table 2:** Summary of Significant Statistical Tests

Dependent Variables	Measures	Independent Variables	N_Laypeople_	N_First Responders_	Mann-Whitney U	Sig.
Tourniquet performance	Time to bleeding control	Group: Laypeople vs. First Responder	24	31	149.50	<.001
	Total application time	Group: Laypeople vs. First Responder	24	31	147.50	<.001
Dependent Variables	Measures	Independent Variables	df	F	Sig.	partial η^2^
CPR Performance	Compression release	Condition: Calm vs. Stressful	1, 45	9.170	.004	.169
Stress (subjective)	Task engagement	Condition: Baseline, Calm, Stressful	1.785, 92.818	6.396	.004	.110
		Planned contrast: Baseline vs. Calm and Stressful	1, 52	7.481	.009	.126
		Planned contrast: Calm vs. Stressful	1, 52	4.068	.049	.073
		Group: Laypeople vs. First Responder	1, 52	5.185	.027	.091
	Distress	Condition: Baseline, Calm, Stressful	2, 102	25.399	<.001	.332
		Planned contrast: Baseline vs. Calm and Stressful	1, 51	46.740	<.001	.478
		Group: Laypeople vs. First Responder	1, 51	22.350	<.001	.305
	Worry	Condition: Baseline, Calm, Stressful	1.548, 75.854	67.418	<.001	.579
		Planned contrast: Baseline vs. Calm and Stressful	1, 49	83.533	<.001	.630
		Planned contrast: Calm vs. Stressful	1, 49	4.762	.034	.089
		Group: Laypeople vs. First Responder	1, 49	6.252	.008	.136
Stress (physiological)	Heart rate	Condition: Calm vs. Stressful	1, 33	190.242	<.001	.852
	LF HRV	Condition: Calm vs. Stressful	1, 30	14.842	<.001	.331
		Group: Laypeople vs. First Responder	1, 30	7.929	.009	.209
	HF HRV	Condition: Calm vs. Stressful	1, 27	5.326	.029	.605
Workload	NASA TLX	Condition by Task interaction	1, 53	4.577	.037	.079
		Group: Laypeople vs. First Responder	1, 53	9.732	.003	.155

*Note*. CPR = cardiopulmonary resuscitation; HRV = heart rate variability; NASA TLX = NASA Task Load Index.

For the CPR task, there was no effect of group or condition on the chest-compression frequency scores or compression depth (all *p* > .177), but there was an effect of condition on compression release scores. The effect was such that all participants had lower proportion of compressions with sufficient release in the stressful condition (*M* = 38.89, SD = 39.54) as compared with the calm condition (*M* = 57.06, SD = 39.79).

### Dundee Stress State Questionnaire

**Figure 2:**
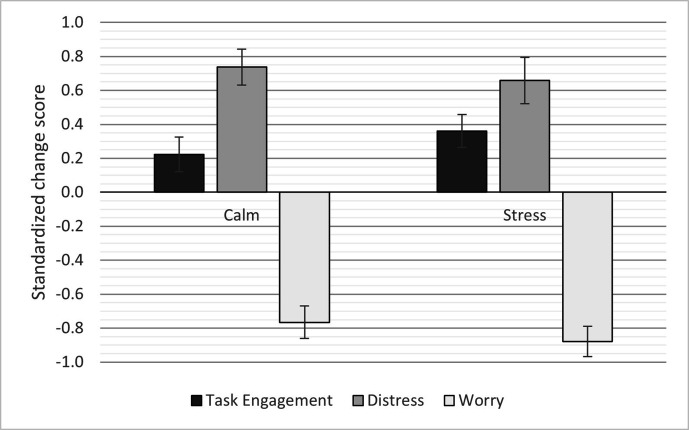
DSSQ change scores across conditions. Error bars show standard errors. *Note*. DSSQ = Dundee Stress State Questionnaire.

The ANOVA on task engagement showed a significant effect of condition, such that the participants overall had higher task engagement during task performance in comparison to the baseline and also higher task engagement during the stressful conditions as compared with the calm condition, see Table 2 and Figure 2. The first responder group also had significantly higher task engagement (*M* = .36, SE = .10) compared with the laypeople group (*M* = .01, SE = .12), see Table 2.

There was also a significant effect of condition on distress, such that the participants overall had higher distress during task performance in comparison to the baseline. However, there was no difference between the stressful conditions and the calm condition, *p* = .388. The first responder group also had significantly lower distress scores overall (*M* = .08, SE = .12) compared with the laypeople group (*M* = .94, SE = .12), see Table 2.

The ANOVA test on worry also showed a significant effect of condition such that the participants overall had lower worry during task performance in comparison to the baseline, and also lower worry during the stressful conditions as compared with the calm condition. The first responder group also had significantly lower worry (*M* = −.72, SE = .10) compared with the laypeople group (*M* = −.31, SD = .11).

### Physiological Response

The ANOVA on mean heart rate showed a significant effect of condition only (see Table 2), such that the participants had significantly higher mean heart rate in the stressful condition (*M* = 134.56, SD = 15.11) as compared with the calm condition (*M* = 97.14, SD = 17.29). This was also the case for HF HRV power, where the stressful conditions had lower values (*M* = 145.24, SD = 124.69) as compared with the calm condition (*M* = 344.79, SD = 421.11). For LF HRV power, there was a significant effect of condition, such that the participants had lower values in the stressful conditions (*M* = 469.87, SD = 508.35) than the calm conditions (*M* = 963.55, SD = 662.18). There was also an effect of group, such that the first responders had overall lower values (*M* = 506.29, SD = 631.64) than the laypeople (*M* = 927.121, SD = 576.47).

### NASA TLX

**Figure 3:**
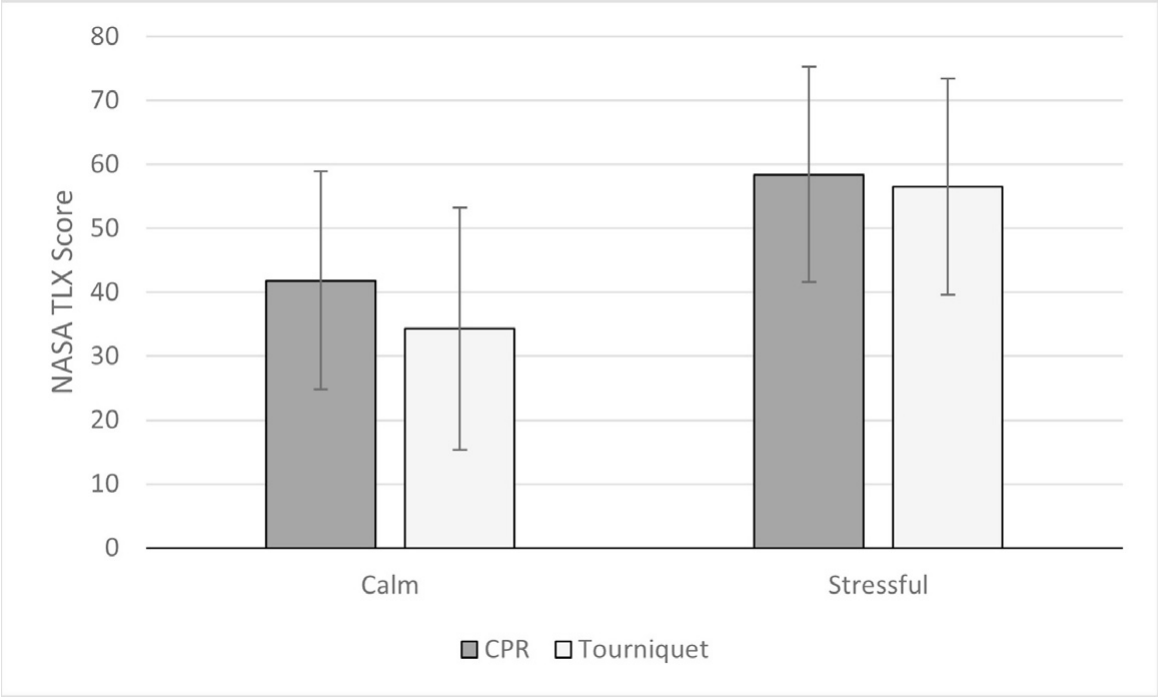
Mean NASA TLX score over condition. Error bars show standard deviation. *Note*. NSA TLX = NASA Task Load Index.

The ANOVA on NASA TLX mean scores showed significant main effects of both condition and task, as well as a significant interaction between condition and task. The interaction was such that the CPR task was associated with higher TLX scores (*M* = 42.43, SE = 2.26) compared with the tourniquet task (*M* = 35.38, SE = 2.32) in the calm condition, *p* = .002, but not in the stressful condition, *p* = .168 (*M* = 59.03, SE = 2.23 and *M* = 57.24, SE = 2.20, respectively; Figure 3).

There was also a significant increase in TLX scores from the calm to the stressful condition for both tasks, *p* < .001. There was also a significant effect of group, see Table 2, such that the first responder group had lower overall TLX scores (*M* = 42.69, SE = 2.47) as compared with the laypeople (*M* = 54.34, SE = 2.80).

## Discussion

Overall, the results showed that the task performance conditions were associated with increased task engagement and distress but lower worry, as compared with the pre-task baseline. The stressful condition was further associated with an increased task engagement, increased heart rate, and decreased HF HRV power as compared with the calm condition. This indicates that the experimental manipulations used in the current study achieved the desired effect of inducing a stress response among the participants.

Further, the first responders had overall higher task engagement, and lower distress and worry as compared with the laypeople. The professional first responders also outperformed the laypeople on the tourniquet task in terms of speed of application and time to bleeding control, but there was no difference in the quality of application as measured by the checklist. Not surprisingly, it thus appears that the professional first responders were able to maintain focus and perform the bleeding control task well in both conditions, more so than the novices.

There was an overall *increase* in tourniquet performance, as measured by the checklist scores, in the stressful condition. This may be due to an ordering effect, as this was the second condition for all participants. It could also potentially be due to increased task focus as indicated by the DSSQ scores. However, some aspects of CPR performance *decreased* for both groups in the stressful condition, as indicated by a lower proportion of compressions with sufficient release. This speaks against a general, positive performance effect from increased task engagement. It is also possible that the CPR task is more sensitive to the increased physiological response compared with the tourniquet task, or it might be an effect of fatigue as performing CPR compressions are physically exhausting. However, a fatigue response would likely also affect the compression depth, not only the release depth. In terms of workload, the CPR was rated as having higher workload during the calm condition as compared with the tourniquet application, which is reasonable given that the CPR task required 90 s of compressions. In the stressful conditions, there was no difference between the tasks, which may derive from the overall increased workload from the physical activity and avoiding paintball fire.

It should also be noted that the physiological response to the stressful condition included an increased heart rate and decreased HF and LF HRV power. Generally, stress is associated with increased heart rate, decreased HF power but also with increased, rather than decreased, LF HRV power (Kim et al., 2018). Some studies have pointed to a differential effect where the LF HRV power is low, rather than high, during particularly stressful conditions (von Rosenberg et al., 2017; see also Kim et al., 2018). This indicates that these physiological measures should be interpreted carefully and in the context of other measures such as the DSSQ.

Although it would have been preferable to counterbalance the order of the two task conditions, this was deemed experimentally difficult given the time required for participants to calm down before performing in the calm condition or the increased sample size necessary for a full between-samples design. Further, the order of performing in a calm environment followed by a stressful environment is the same as in Schreckengaust et al. (2014). It would also have been preferable to have a more even balance between genders in the recruited sample, although the first responder professions are still male-dominated populations.

Another limitation of the current study is the stress manipulation and its generalizability to real-world settings. The use of paintball, fear of pain and physical exhaustion mirror Schreckengaust et al.’s (2014) manipulation and thus validates their method in terms of a provocation of stress effects. However, the stress profile that resulted, with increased task engagement and distress and reduced worry, may be very different from the stress profile experienced during a real emergency. Without a doubt, there are cases where a direct threat to the immediate or first responders’ own person and physical well-being is the primary driving stressor, such as in cases of ongoing antagonistic attacks or in presence of other environmental hazards. However, Kindness et al. (2014) and Bohström et al. (2017) show that stressors such as feelings of lack of control, frustration with lack of adequate resources, fear of an anticipated negative outcome (e.g., patient death), and feelings of inadequacy, isolation, and loneliness are also primary drivers of experienced stress among prehospital first responders and immediate responders. It is plausible that such stressors would generate a qualitatively different stress profile and other coping mechanisms and subsequent performance effects among the responders. These types of stressors need to be further explored in controlled settings, to the extent that is possible.

In summary, the results show that the stress manipulation of paintball fire and physical effort affected the participants but also that this only impaired some aspects of CPR performance and not tourniquet application performance. Further, the first responders performed better than the novices on the tourniquet application task regardless of condition and showed fewer stress reactions. As CPR is something commonly taught in first aid classes for laypeople, it seems reasonable to conclude, based on these results, that bleeding control could also feasibly be taught. It might also be beneficial to include some form of stress management or stress exposure training in such first aid courses, as such stress trainings are likely to generalize to novel stressors encountered during real emergencies (Driskell et al., 2001). Further, even though tourniquet performance was not degraded by stress, there was still a difference between the two groups where the professional first responders outperformed the laypeople. This indicates that although laypeople may be able to perform basic skills after a brief training intervention, there are performance benefits for more rigorous training to, for example, reduce application times.

A concern raised in the previous literature (e.g., Goolsby, Strauss-Riggs, et al., 2018) is that novices may not be able to perform bleeding control interventions well under stress. The current study instead shows that this should not be a primary concern in the decision whether to teach bleeding control skills to laypeople, given that CPR, which is already widely taught, appears to be more sensitive to performance decrements. It would be more productive to orient current first aid education research toward the question of how to best prepare and train laypeople to perform effectively under stress.

## Key Points

- An argument against including hemorrhage control skills in first aid courses for laypeople is that laypeople may underperform in stressful settings. However, this has not been investigated empirically.
- The current study shows that CPR is more sensitive for performance decrements in stressful conditions than tourniquet application.
- Laypersons can be trained to effectively apply tourniquets and their performance immediate post-training does not deteriorate in stressful conditions, similarly to professional first responders.
